# Multiple giant placental chorioangioma: A case report

**DOI:** 10.1002/ccr3.9219

**Published:** 2024-07-25

**Authors:** Atefe Hashemi, Shaghayegh Moradi Alamdarloo, Homeira Vafaei, Hamide Barzegar, Firouzeh Jafari, Sara Haseli, Elahe Abbaspour

**Affiliations:** ^1^ Maternal‐Fetal Medicine Research Center, Department of Obstetrics and Gynecology, School of Medicine Shiraz University of Medical Sciences Shiraz Iran; ^2^ Neonatal Research Center Shiraz University of Medical Sciences Shiraz Iran; ^3^ Department of Pathology, School of Medicine Shiraz University of Medical Sciences Shiraz Iran; ^4^ Department of Radiology, Division of Musculoskeletal Imaging and Intervention University of Washington Seattle Washington USA; ^5^ The OncoRad Research Core, Department of Radiology University of Washington/Fred Hutchinson Cancer Center Seattle Washington USA; ^6^ Department of Radiology, Poursina Hospital Guilan University of Medical Sciences Rasht Iran

**Keywords:** case report, Doppler ultrasound, giant chorioangioma, placental chorioangioma, placental tumor

## Abstract

**Key Clinical Message:**

Giant chorioangiomas, despite being rare, pose significant fetal and maternal risks. Timely and individualized treatment plans are crucial to reduce morbidity and mortality when fetal compromise occurs. Additionally, successful conservative management relies on consistent ultrasound monitoring, Doppler flowmetry assessments, and amniotic fluid level measurements.

**Abstract:**

Chorioangiomas are benign placental tumors that manifest in approximately 1% of pregnancies. Giant chorioangiomas, characterized by tumors exceeding 4 cm, are exceptionally rare and pose substantial risks to maternal and fetal health. This case report details a patient with multiple giant chorioangiomas, emphasizing the rarity and consequential complications associated with these tumors. A 23‐year‐old woman, G3P2, at 28 weeks gestational age, was diagnosed with multiple large, well‐defined placental masses with increased vascularity, indicative of giant placental chorioangiomas. Subsequent ultrasound revealed various fetal anomalies such as cleft palate and lip, as well as lung and heart abnormalities. At 34^+5^ weeks of gestation, an emergency cesarean section was performed due to preeclampsia. Subsequently, a female neonate was born with hydrops fetalis. Unfortunately, she passed away within the first hour of her life. Complications associated with chorioangiomas primarily arise from arteriovenous shunts, which potentially lead to compromised fetal perfusion and cardiac failure. Although small‐sized chorioangiomas are often discovered incidentally, Doppler ultrasound and magnetic resonance imaging can reliably distinguish these tumors from other placental lesions. Additionally, management strategies tailored to gestational age and maternal‐fetal symptoms typically necessitate a multidisciplinary approach. However, additional research is essential to understand the mechanisms of chorioangiomas and to develop comprehensive management guidelines.

## Graphical abstract

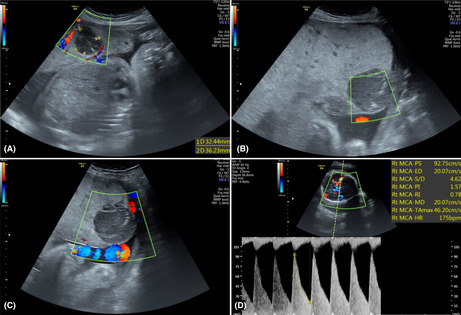

## 1 INTRODUCTION

Chorioangioma is a benign vascular tumor of the placenta that occurs in approximately 1% of pregnancies.^1^ The majority of cases are small and asymptomatic, with symptoms appearing in only 0.01%–0.03% of instances.^2^ Giant chorioangiomas, defined as tumors larger than 4 cm, are remarkably rare, with a prevalence ranging from 1:9000 to 1:50,000.^3^ While many chorioangiomas are detected during postnatal examination of placental histology, large chorioangiomas are associated with significant maternal and fetal complications. These include preterm labor, fetal growth restriction (FGR), preeclampsia, polyhydramnios as well as hydrops fetalis, disseminated intravascular coagulation (DIC), and mortality.^4^, ^5^, ^6^

Despite significant advancements in therapeutic approaches, perinatal mortality rates remain high, estimated to be more than 30%.^7^ Therefore, it is essential to highlight the importance of timely identification, comprehensive prenatal monitoring, and appropriate interventions to prevent fetal morbidity and mortality.^8^ In this report, we present a case involving multiple giant chorioangiomas in a 23‐year‐old woman, which were associated with fetal complications and ultimately resulted in the neonate's death due to hydrops fetalis. This case emphasizes the complexity of this condition and underscores the necessity for a multidisciplinary approach in evaluating and counseling patients with intricate fetal anomalies. This study has been reported in line with the CARE criteria.^9^

## 2 CASE HISTORY/EXAMINATION

A 23‐year‐old Afghan woman (gravida 3 para 2) at 31 weeks of gestation presented to our clinic following a routine ultrasound examination at 28 weeks that had raised concerns regarding placental abnormalities. The patient had previously undergone two cesarean sections (CS) and had an unremarkable medical or familial history. Her first pregnancy, 4 years prior, ended with a preterm cesarean section at 36 weeks due to ruptured membranes and breech presentation. During her second pregnancy, she developed gestational diabetes, which was successfully managed with metformin, ultimately resulting in a term delivery. The current pregnancy progressed uneventfully. However, due to her poor socioeconomic status, the patient did not receive appropriate prenatal care and did not undergo the first or second‐trimester screening tests or the fetal anomaly scan. The initial ultrasound at 19 weeks revealed an intrauterine gestation consistent with dates, with the placenta forming anteriorly. The follow‐up scan at 28 weeks showed multiple masses within the placenta, along with increased placental thickness (77 mm), suggesting chorioangiomas or placental cysts as likely diagnoses. Despite these findings, fetal growth remained satisfactory, with no evidence of cardiovascular compromise and a normal amniotic fluid index (AFI = 20). Additionally, the patient's glucose tolerance test results were as follows: fasting glucose was 72, 1‐hour post‐test was 166, and 2‐hour post‐test was 127. These results indicated negative findings, suggesting the absence of gestational diabetes. Considering the complexity of the case, the patient was referred for further evaluation.

### 2.1 Differential diagnosis, investigations, and treatment

**FIGURE 1:**
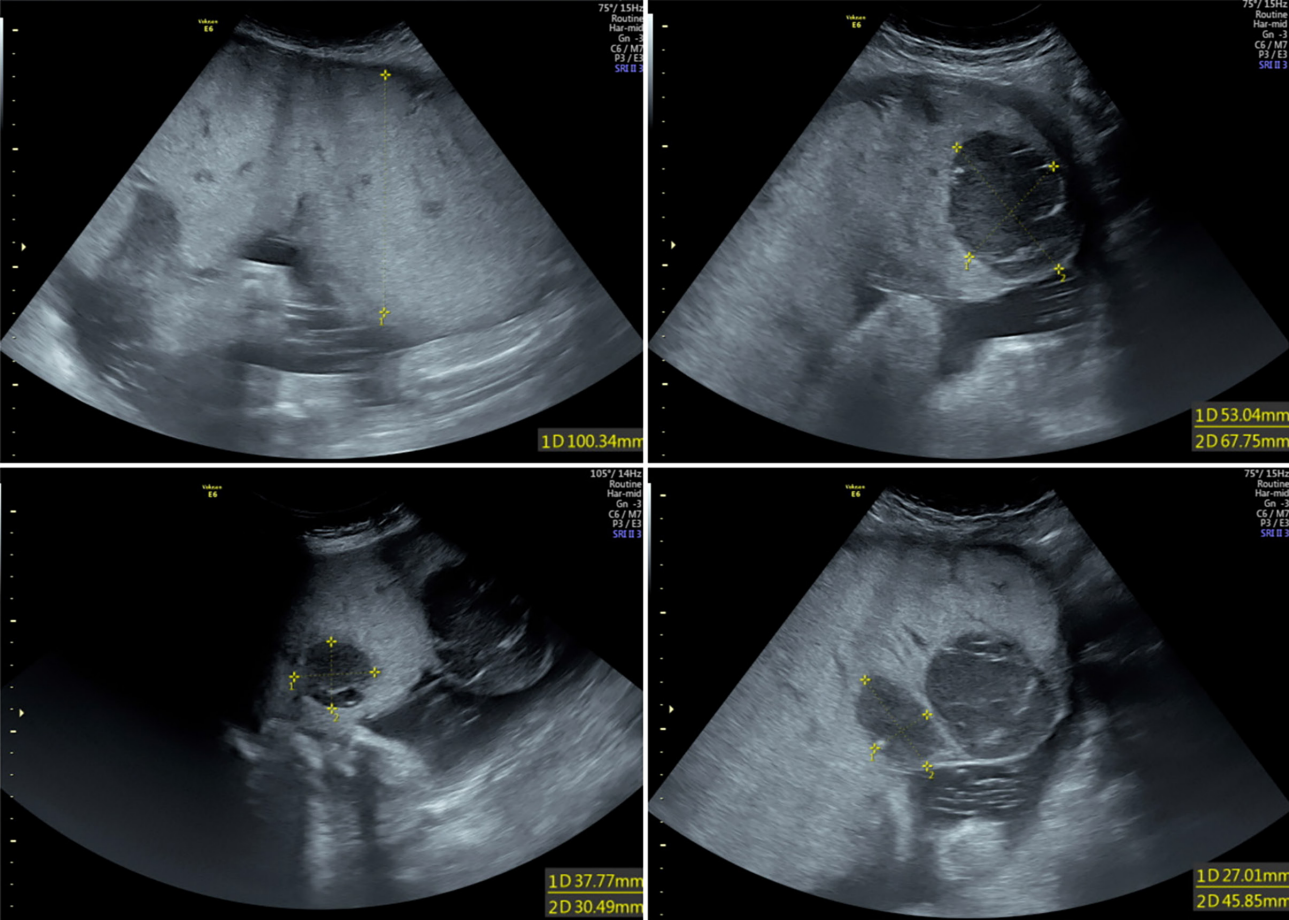
The ultrasound reveals six distinct hypoechoic lesions within the placenta.

**FIGURE 2:**
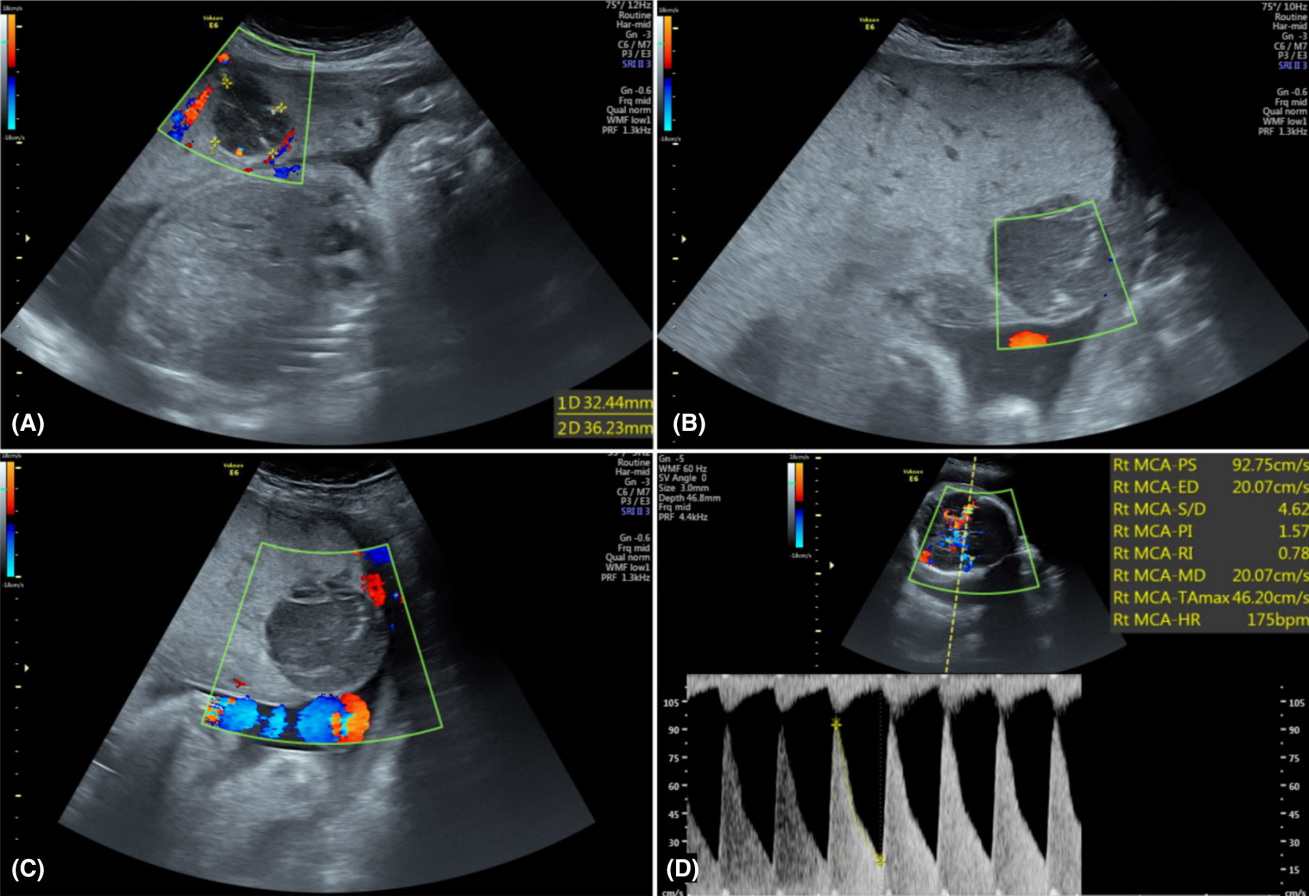
(A–C) The Doppler ultrasound of placental chorioangiomas showing the feeding vessels. (D) The MCA‐PSV measurement at 31 weeks of gestation corresponded to 2.14 multiples of the median (MoM).

We performed a fetal ultrasound, which revealed multiple well‐defined hypoechoic lesions within the placenta with increased vascularity, indicating placental chorioangioma (Figure 1). Additionally, various fetal anomalies were identified, including features indicative of large for gestational age, cleft lip and palate, hypoplastic lung, and a hyperechoic lung mass adjacent to the left atrium with systemic circulation. Notably, no signs of hydrops fetalis were observed. Moreover, fetal Doppler assessment revealed an increased peak systolic velocity of the middle cerebral artery (MCA‐PSV) measuring 92, with 2.14 multiples of the median (MoM) for the gestational age, and an umbilical artery pulsatility index (PI) of 1.01. These findings were indicative of fetal anemia, accompanied by pulsatile turbulent flow (Figure 2).

Following thorough parental counseling regarding the risks of maternal and fetal complications along with the management of anemia, she was advised to attend weekly follow‐up appointments to monitor the severity of the condition and assess the need for intrauterine transfusion. However, the patient declined invasive treatments and chose not to attend subsequent follow‐up visits.

### 2.2 Outcome and follow‐up

At 34^+5^ weeks gestation, the patient presented to our emergency department with complaints of labor pain. The subsequent workups revealed the diagnosis of preeclampsia, prompting the decision for emergency CS delivery. A female neonate weighing 2315 g was delivered, measuring 45 cm in length and with a head circumference of 32 cm. The neonate exhibited Appearance, Pulse, Grimace, Activity, and Respiration (APGAR) scores of 1 at 1 and 5 min and was diagnosed with hydrops fetalis. Unfortunately, despite resuscitative efforts, the neonate passed away within the first hour of life.

**FIGURE 3:**
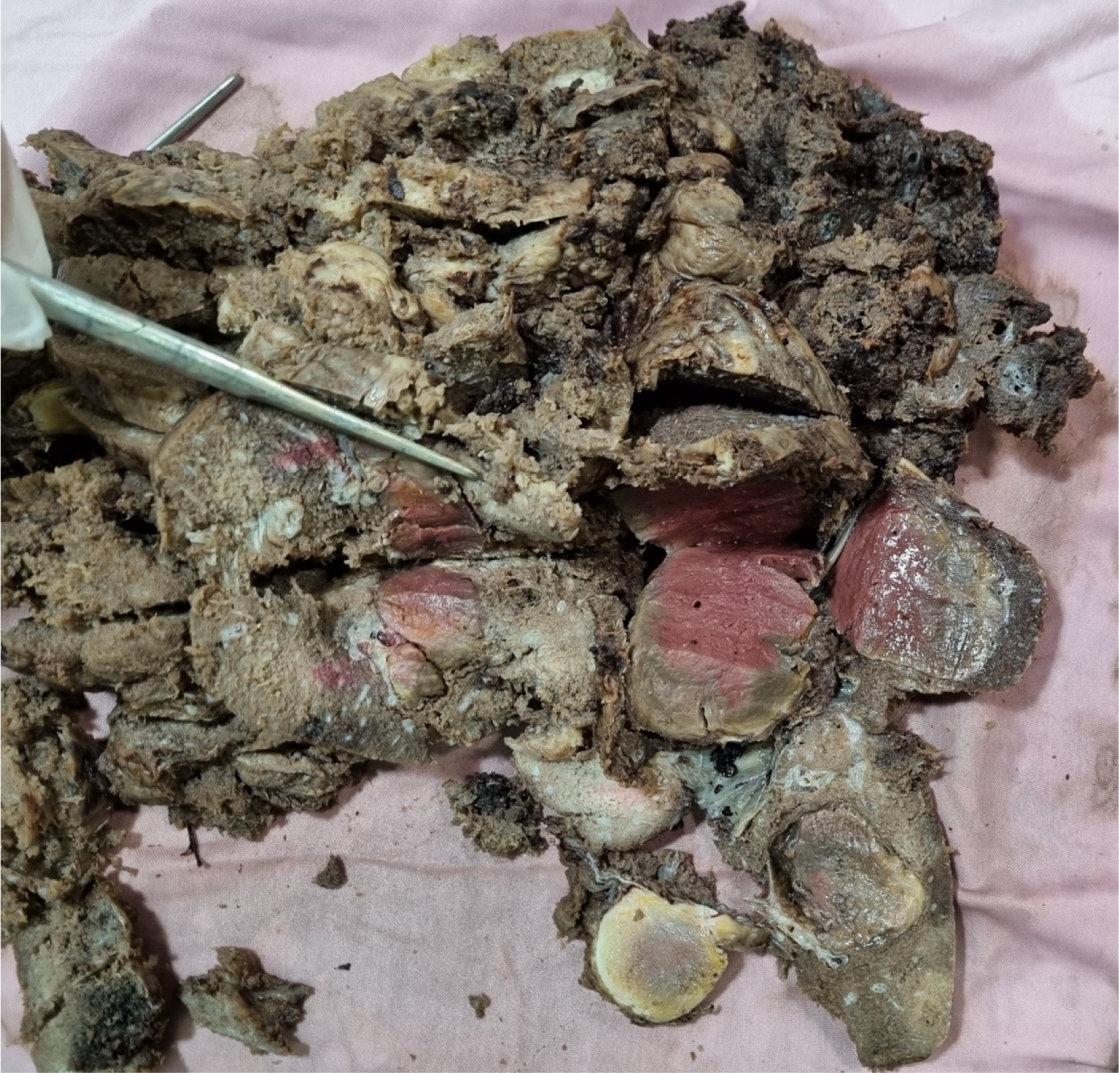
The macroscopic examination of the placenta reveals several distinct, firm, and rubbery nodules situated on the chorionic plate, with some exhibiting signs of recent or old infarction.

**FIGURE 4:**
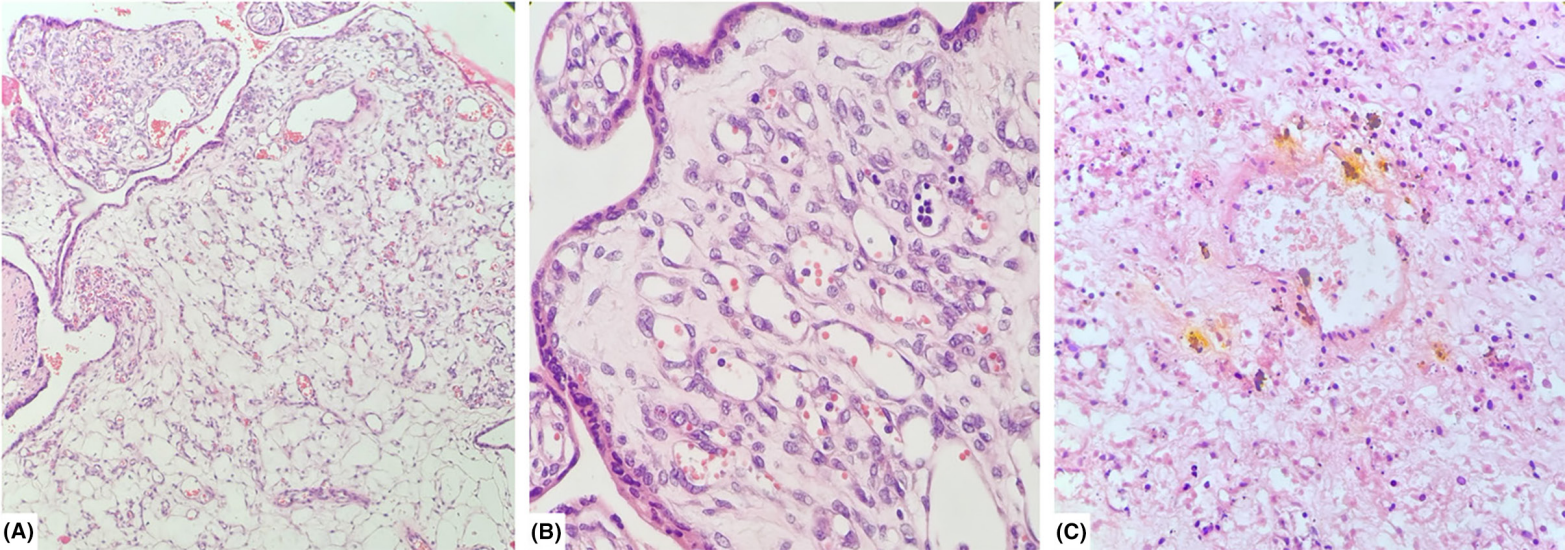
(A) Microscopic examination shows numerous capillary‐sized vascular channels covered by a trophoblastic layer (H&E × 10). (B, C) Infarction and hemosiderin deposition can be seen (H&E × 40).

The placenta weighed 1300 g and measured 26 cm in greatest diameter, with a maximum thickness of 9 cm at the central portion. The umbilical cord measured 24 cm in length. Upon sectioning, multiple well‐defined creamy‐red rubbery tissues were observed, with the largest masses measuring 6 × 5 × 4 cm, 4.2 × 4 × 3 cm, and 4 × 3.5 × 3.5 cm along the chorionic surface of the placenta (Figure 3). The retroplacental hemorrhage covered approximately 10% of the placental surface area. The histopathological examination revealed a distinctive pattern of trophoblastic layers enveloping numerous capillary‐sized vascular channels. Additionally, focal laminar decidual necrosis and the presence of meconium‐laden macrophages were noted, conclusively confirming the diagnosis of placental chorangioma, capillary type (Figure 4). The patient's recovery remained uneventful, with no reported postoperative complications.

## 3 DISCUSSION

Placental chorioangioma is a nontrophoblastic tumor originating from primitive chorionic mesenchyme.^10^ While the definitive cause of the condition remains elusive, various risk factors have been associated with its onset, including primiparity, maternal age exceeding 30, multifetal gestation, maternal hypertension, and diabetes, as well as carrying a female fetus.^11^, ^12^ Our case aligned with these findings as it involved multifetal gestation, high blood pressure, preeclampsia, and a female fetus.

Small‐sized chorioangiomas are often incidentally discovered during the third trimester, whereas larger tumors (>4 cm) are more easily identified.^13^ The diagnosis primarily relies on ultrasound, where they appear as well‐defined lesions with distinct echogenicity compared to surrounding tissue.^11^, ^14^, ^15^ They are commonly found in close proximity to the cord insertion site on the fetal surface of the placenta, where they extend into the amniotic cavity.^16^ Furthermore, color Doppler ultrasound is instrumental in distinguishing chorioangiomas from other placental lesions such as placental hematomas, chorangiocarcinoma, partial hydatidiform moles, teratomas, and degenerated fibroids.^10^, ^17^ Moreover, it can reveal the presence of a prominent feeding vessel within the tumor or confirm the vascular continuity between the tumor and fetal circulation.^18^ Magnetic resonance imaging (MRI) commonly shows chorioangiomas as heterogeneous masses with iso‐intensity to the placenta on T1‐weighted images and hyper‐intensity on T2‐weighted images.^15^, ^16^ Additionally, MRI offers further anatomical information regarding the tumor's extent and its connections with nearby structures, which can be beneficial in intervention planning.^19^, ^20^, ^21^

Placental chorioangiomas exhibit three histological subtypes. The capillary type is characterized by numerous capillary enlargements, the cellular type by dense parenchyma of immature cells with fewer angiogenesis, and the degenerative type by diverse degenerative changes, including fibrinoid necrosis and calcification.^16^, ^22^ The vascularization of chorioangiomas is a prognostic factor affecting pregnancy outcomes. While nonvascularized chorioangiomas are generally uncomplicated, vascularized tumors can lead to various complications.^23^ Polyhydramnios is the most common maternal complication, occurring in 18%–35% of cases of giant chorioangiomas.^10^ In addition, the primary fetal complications include hemolytic anemia, thrombocytopenia, nonimmunologic hydrops, congenital anomalies, cardiomegaly, and congestive heart failure.^16^ The pathophysiological mechanism underlying these complications arises from the presence of vascular channels within the tumor, which act as arteriovenous shunts. These shunts compromise the perfusion of chorionic villi, leading to reduced delivery of essential nutrients and oxygen to the fetal circulation. As a result, the fetal heart compensates to maintain tissue perfusion.^1^, ^7^, ^11^ However, inadequate compensation may result in cardiac failure and hydrops fetalis, which often leads to adverse outcomes and fetal mortality,^3^ as observed in our case.

The management of pregnancy complications depends on both gestational age and maternal‐fetal symptoms.^14^ A promising therapeutic approach for managing symptomatic cases involves blocking the tumor‐feeding vessels. Various methods for achieving this objective include fetoscopy‐assisted laser ablation or suture ligation, alcohol injection, microcoil embolization, and ultrasound‐guided embolization.^3^, ^24^, ^25^ The selection of the most appropriate vessel occlusion technique depends on factors such as the anatomical location or diameter of the target vessel and its proximity to the umbilical cord insertion site.^2^ Conservative management is feasible by closely monitoring the chorioangioma's vascularity and growth as well as progressive fetal distress.^15^ Regular monitoring can extend gestation and prevent fatal complications through the timely use of intrauterine fetal transfusion and embolization of the supplying vessel.^26^, ^27^ This highlights the potential for a positive fetal and maternal outcome with systematic follow‐up.

## 4 CONCLUSION

Placental chorioangiomas displaying signs of fetal compromise often result in adverse perinatal outcomes. Successful conservative management requires consistent ultrasound monitoring, Doppler flowmetry assessment, and measurement of amniotic fluid levels. An attentive clinical approach assists in the timely identification of potential vascular masses and reduces the risk of severe complications. However, further research is necessary to explore chorioangioma mechanisms and establish comprehensive management guidelines.

## AUTHOR CONTRIBUTIONS

**Atefe Hashemi:** Data curation; investigation; writing – original draft. **Shaghayegh Moradi Alamdarloo:** Data curation; investigation; writing – original draft. **Homeira Vafaei:** Conceptualization; methodology; visualization; writing – review and editing. **Hamide Barzegar:** Conceptualization; methodology; visualization; writing – review and editing. **Firouzeh Jafari:** Conceptualization; methodology; validation; writing – original draft. **Sara Haseli:** Project administration; resources; supervision; validation; writing – review and editing. **Elahe Abbaspour:** Project administration; supervision; validation; writing – review and editing.

## FUNDING INFORMATION

None.

## CONFLICT OF INTEREST STATEMENT

The authors declare that they have no competing interests.

## ETHICAL APPROVAL

The institutional board review at Shiraz University of Medical Sciences granted approval for the study is available upon request from the corresponding author (Ethics approval code: IR.SUMS.REC.1402.580).

## CONSENT

Written informed consent was obtained from the patient to publish this report in accordance with the journal's patient consent policy.

## Data Availability

The data used to support the findings of this case report are available from the corresponding author upon request. Anonymized and aggregated data that do not compromise patient confidentiality can be made available to researchers for further analysis upon request and appropriate ethical approval.
